# Patient versus general population health state valuations: a case study of non-specific low back pain

**DOI:** 10.1007/s11136-017-1497-5

**Published:** 2017-02-02

**Authors:** J. M. van Dongen, B. van denBerg, G. E. Bekkering, M. W. van Tulder, R. W. J. G. Ostelo

**Affiliations:** 1 Department of Health Sciences and the EMGO+ Institute for Health and Care Research, VU University, De Boelelaan 1085, 1081 HV Amsterdam, The Netherlands; 2 University of York, Centre for Health Economics, University of York, Heslington, York, YO10 5DD UK; 3 Faculty of Economics and Business, University of Groningen, Nettelbosje 2, 9747 AE Groningen, The Netherlands; 4 CEBAM Belgian Center of Evidence-based Medicine vzw, Kapucijnenvoer 33, blik j, 3000 Leuven, Belgium; 5 Department of Epidemiology and Biostatistics and the EMGO+ Institute for Health and Care Research, VU University Medical Centre, De Boelelaan 1089a, 1081 HV Amsterdam, The Netherlands

**Keywords:** Low back pain, Health state valuation, Cost-effectiveness analysis, EQ-5D-3L, EQ-VAS

## Abstract

**Purpose:**

The purpose of this study was twofold: (1) to compare non-specific low back pain (LBP) patients’ health state valuations with those of the general population, and (2) to explore how aspects of health-related quality of life as measured by the EQ-5D-3L impact non-specific LBP patient valuations.

**Methods:**

Data were used of a randomized controlled trial, including 483 non-specific LBP patients. Outcomes included the EQ-VAS and the EQ-5D-3L. Patient valuations were derived from the EQ-VAS. Population valuations were derived from the EQ-5D-3L using a Dutch VAS-based tariff. The difference between patient and population valuations was assessed using *t* tests. An OLS linear regression model was constructed to explore how various aspects of health-related quality of life as measured by the ED-5D-3L impact non-specific LBP patient valuations.

**Results:**

Non-specific LBP patients valued their health states 0.098 (95% CI 0.082–0.115) points higher than the general population. Only 22.2% of the variance in patient valuations was explained by the patients’ EQ-5D-3L health states (*R* ^2^ = 0.222). Non-specific LBP patients gave the most weight to the anxiety/depression dimension.

**Conclusions:**

This study demonstrated that non-specific LBP patients value their health states higher than members of the general population and that the choice of valuation method could have important implications for cost-effectiveness analyses and thus for clinical practice.

## Introduction

The high disease and economic burden of low back pain (LBP) has led to the development and evaluation of a broad range of LBP treatments [1–3]. Cost-effectiveness analyses can support decision-makers on the allocation of scarce resources by comparing alternative (LBP) treatments in terms of both their costs and effects [4]. Quality-adjusted life years (QALYs) are often used as an effect measure in cost-effectiveness analyses. QALYs capture the two most important features of a treatment in one metric; i.e., its effect on *quantity* of life and its effect on *quality* of life. For estimating a treatment’s effect on *quality* of life, health state valuations are used. Health state valuations are basically preference weights, indicating a person’s preference for a health state on a scale anchored at 0 (equal to death) and 1 (equal to full health) [4, 5].

Even though the valuation of health states is essential for cost-effectiveness analyses, debate still exists as to whom should value health states [4–6]. That is, whether health states should be valued by patients themselves or by members of the general population. In most studies, members of the general population are used [6, 7]. Others argue that patient valuations should be used [6, 8].

To our knowledge, Mann et al. have been the only ones to compare patient and population valuations among non-specific LBP patients (i.e., LBP with no specific cause, such as a tumor, infection, fracture, and herniated disk) [9]. They found patient valuations to be lower than population valuations, indicating that non-specific LBP patients perceive their health states to be worse than the general population does. This, however, contrasts the results of other studies in this area, most of which found patient valuations to be higher than population valuations, or to be similar [10, 11]. Therefore, the aim of the current study was twofold: (1) to compare non-specific LBP patients’ health state valuations with those of the general population and (2) to explore how various aspects of health-related quality of life as measured by the EQ-5D-3L impact non-specific LBP patient valuations.

## Methods

### Design

Data were used of a randomized controlled trial (RCT) assessing the (cost-)effectiveness of an active implementation strategy for the Dutch Physical Therapy guidelines for LBP patients [12]. The study was approved by the Medical Ethics Committee of the VU University Medical Centre, Amsterdam, the Netherlands.

### Study population

The study population consisted of 483 Dutch non-specific LBP patients. They were referred by their general practitioner to a physical therapist to receive treatment for a new episode of non-specific LBP [12]. The participants’ baseline characteristics are provided in Table 1.

**Table 1:** Baseline characteristics of the study population

Baseline characteristics	Low back pain patients (*n* = 483)
Age [mean (SD)]	44.4 (14.0)
Gender [*n* (%); female]	250 (51.8)
Pain [mean (SD); Numerical Rating Scale 0–10]	6.3 (2.0)
Prior episode of LBP [*n* (%); yes]	351 (72.7)
Duration current LBP episode [*n* (%)]	
0–6 weeks (acute non	241 (49.9)
6–12 weeks (sub	85 (17.6)
>12 weeks (chronic non-specific LBP)	151 (31.3)
Unknown	6 (1.2)
Paid job [*n* (%); yes]	353 (73.1)
Sick-leave [*n* (%)]	
Yes	154 (31.9)
No	193 (40.0)
Not applicable	131 (27.1)
Unknown	5 (1.0)

*SD* standard deviation, *n* number, *LBP* low back pain

### Outcome measures

Outcome measures included the EQ-VAS and the EQ-5D-3L. In the RCT, both were measured at baseline, 6, 12, 26, and 52 weeks. For this study, we solely used baseline data, where EQ-VAS and EQ-5D-3L data were complete.

#### EQ-VAS

The EQ-VAS is a visual analogue scale, ranging from ‘worst imaginable health state’ (0) to ‘best imaginable health state’ (100).

#### EQ-5D-3L

The EQ-5D-3L consists of five health dimensions: (1) mobility; (2) self-care; (3) usual activities; (4) pain/discomfort; and (5) anxiety/depression. Each dimension contains three severity levels: (1) no problems; (2) some or moderate problems; and (3) severe problems. For each dimension, patients are asked to report the level that best describes their current health state. The five health dimensions combined with the three severity levels result in 243 possible health states, ranging from 11111 (no problems on all dimensions) to 33333 (severe problems on all dimensions) [13].

#### Estimating patient valuations

The participants’ EQ-VAS scores were transformed into health state values, where 0 represents death and 1 represents full health. Note that simply dividing EQ-VAS scores by 100 will not achieve this, as an EQ-VAS score of 0 is not necessarily equivalent to death and an EQ-VAS score of 100 is not necessarily equivalent to full health [14]. Therefore, EQ-VAS scores were transformed into health state values using the following formula (further referred to as patient valuations):

$$\text{Patient valuations}=(\text{VAS}_\text{score}/100)-\text{VAS}_\text{death})/(\text{VAS}_{11111}-\text{VAS}_\text{death}),$$

where VAS_death_ is assumed to equal 0.085 and VAS_11111_ 0.987 [15].

#### Estimating population valuations

The participants’ EQ-5D-3L health states were converted into health state values using a Dutch VAS-based tariff (further referred to as population valuations) [14]. The Dutch VAS-based tariff was developed using a sample of 212 adults that were representative of the Dutch population with regard to age, gender, and perceived health. Within this study, participants were asked to rate a number of EQ-5D-3L health states on a VAS scale. Using these data, the authors developed an algorithm (i.e., tariff) that can be used to convert EQ-5D-3L health states into health state values that are based on the preferences of the general population [14].

### Statistical analyses

#### Comparing non-specific LBP patient valuations and population valuations.

Non-specific LBP patient valuations and population valuations were compared using a paired *t* test. To explore whether the difference between patient and population valuations systematically increased with LBP duration, subgroup analyses were performed among acute (LBP for 0–6 weeks), sub-acute (LBP for 6–12 weeks), and chronic LBP patients (LBP for >12 weeks).

#### Relationship between EQ-5D-3L health states and non-specific LBP patient valuations.

To explore how various aspects of health-related quality of life as measured by the EQ-5D-3L impact non-specific LBP patient valuations, an ordinary least squares (OLS) linear regression model was constructed with 1 minus the participants’ patient valuations as dependent variable and 11 independent variables describing their EQ-5D-3L health states. Ten out of the 11 independent variables comprised dummy variables for the EQ-5D-3L dimensions, with the level reflecting “no problems” as reference. In line with the previous models, an N3 dummy variable was added to the model, indicating whether at least one dimension was at level 3 (i.e., 11th independent variable) [14]. The model’s R^2^ was estimated and the coefficients derived from the OLS linear regression model were compared with those of the general population [16].

## Results

### EQ-5D-3L health states

An overview of the frequencies with which the three levels of the EQ-5D-3L dimensions were scored is provided in Table 2. Most notably, patients hardly scored level 3 (‘severe problems’). An overview of all occurring EQ-5D-3L health states and their frequencies can be found in Appendix 1.

**Table 2:** Frequencies with which the three levels of the EQ-5D-3L dimensions were scored

	Mobility (%)	Self-care (%)	Usual activities (%)	Pain/discomfort (%)	Anxiety/depression (%)
Level 1	52.4	75.6	22.2	11.4	77.6
Level 2	47.2	24.2	69.8	79.5	21.3
Level 3	0.4	0.1	8.0	9.1	1.1

### Comparing non-specific LBP patient valuations and population valuations

The participants’ patient valuations (mean = 0.731; SD = 0.172) were statistically significantly higher than their population valuations (mean = 0.632; SD = 0.167) (*β* = 0.098; 95% CI 0.082–0.115). Participants with acute, sub-acute, and chronic non-specific LBP valued their health state significantly higher compared with members of the general population. The discrepancy between patient and population valuations was most pronounced among participants with acute non-specific LBP, and least pronounced among participants with sub-acute non-specific LBP (Table 3).

**Table 3:** mean patient valuations, mean population valuations, and mean differences between patient and population valuations

	Patient valuations Mean (SD)	Population valuations Mean (SD)	Mean difference between patient and population valuations Mean (95% CI)
All non-specific LBP patients (*n* = 483)	0.731 (0.172)	0.632 (0.167)	0.098 (0.082 to 0.115)
Acute non-specific LBP patients (*n* = 241)	0.724 (0.164)	0.608 (0.170)	0.115 (0.092 to 0.139)
Sub-acute non-specific LBP patients (*n* = 85)	0.718 (0.199)	0.656 (0.170)	0.059 (0.020 to 0.097)
Chronic non-specific LBP patients (*n* = 151)	0.748 (0.155)	0.656 (0.155)	0.092 (0.064 to 0.120)

*SD* standard deviation, *n* number, *LBP* low back pain

### Relationship between EQ-5D-3L health states and non-specific LBP patient valuations

Coefficients for the usual activities, pain/discomfort, and anxiety/depression dimensions of the EQ-5D-3L were in the expected direction with larger decrements associated with the higher severity levels, whereas those of the mobility and self-care dimension were not. Five coefficients were statistically significant; all others were not. The model’s *R* ^2^ was 0.222 (Table 4).

**Table 4:** General population and non-specific low back pain patient coefficients for the EQ-5D-3L dimensions

	Definitions	Coefficients General population Mean*	Coefficients Non-specific LBP patients Mean (95%CI) *
Constant	Subtract if at least one level is at 2 or 3	0.173	0.146 (0.103 to 0.190)
Mobility (2)	Subtract if mobility is at level 2	0.101	0.039 (0.008 to 0.069)
Mobility (3)	Subtract if mobility is at level 3	0.227	0.022 (−0.295 to 0.340)
Self-care (2)	Subtract if self-care is at level 2	0.081	0.017 (−0.018 to 0.051)
Self-care (3)	Subtract if self-care is at level 3	0.090	−0.308 (−0.765 to 0.150)
Usual activities (2)	Subtract if usual activities is at level 2	0.040	0.038 (0.001 to 0.074)
Usual activities (3)	Subtract if usual activities is at level 3	0.080	0.088 (−0.016 to 0.192)
Pain / discomfort (2)	Subtract if pain/discomfort is at level 2	0.065	0.038 (−0.006 to 0.083)
Pain / discomfort (3)	Subtract if pain/discomfort is at level 3	0.173	0.074 (−0.036 to 0.184)
Anxiety / depression (2)	Subtract if anxiety/depression is at level 2	0.054	0.085 (0.051 to 0.119)
Anxiety / depression (3)	Subtract if anxiety/depression is at level 3	0.155	0.143 (0.004 to 0.281)
Any dimension at level 3	Subtract if any dimension is at level 3	0.142	0.073 (−0.037 to 0.184)
*R* ^*2*^		0.384	0.222
*n*		298	483

*R* ^*2*^ *R*-squared, *n* number

*Note that both models were constructed with 1 minus the participants’ patient valuations (derived using the EQ-VAS) as dependent variable and 11 independent variables describing their EQ-5D-3L health states

As very few participants scored level three on the mobility and self-care dimension (Table 2), the level 3 coefficients of the mobility and self-care dimension were not compared with those of the general population. When comparing all other coefficients, the usual activities and anxiety/depression coefficients were similar in both models, whereas the decrements for mobility (only level 2), self-care (only level 2), and pain/discomfort were smaller in the non-specific LBP patient model compared with the general population model (Table 4).

## Discussion

### Main findings

#### Comparing non-specific LBP patient valuations and population valuations

This study indicated that non-specific LBP patients value their health states on average 0.098 (95% CI 0.082–0.115) points higher than members of the general population. This difference exceeds the minimal clinically important difference for the EQ-5D-3L (i.e., 0.05–0.08) [16] and is in line with the most recent review on this topic [11]. It should be noted, however, that non-specific LBP patients were not included in this review. In addition, the results of the only study that did include non-specific LBP patients contrast those of the present study [10]. The latter may be due to the absence of acute non-specific LBP patients in the previous study [10] as well as differences between both studies with regard to the percentage of female participants (i.e., 53% in the present study versus 61% in the previous one) as well as the percentage of participants that experienced a prior LBP episode (i.e., 75 versus 84%) [10]. Furthermore, even though clinical experience suggests that people adapt to longer term ill health [4], no evidence was found in the present study to support this proposition. That is, the difference between patient and population valuations did no systematically increase with LBP duration.

#### Relationship between EQ-5D-3L health states and non-specific LBP patient valuations

Only 22.2% of the variance in the participants’ patient valuations was explained by their EQ-5D-3L health states. This indicates that the EQ-5D-3L health state descriptions may be too coarse to comprehensively describe non-specific LBP patient health states and thus their self-perceived health-related quality of life [17]. The recently developed five-level version of the EQ-5D (i.e., EQ-5D-5L) may provide a possible means for improvement, but further research is needed to establish this. It might also be, however, that health dimensions other than those covered by the EQ-5D-3L play an important role in predicting non-specific LBP patients’ self-perceived health-related quality of life (e.g., vitality, well-being, and role functioning). As existing health state questionnaires (e.g., EQ-5D, SF-6D, HUI) vary widely in terms of health dimensions [4, 6], future research is warranted to explore which of them is most suitable for LBP research and clinical practice, and/or whether an LBP-specific health state questionnaire ought to be developed. Results also indicated that non-specific LBP patients gave the most weight to the anxiety/depression dimension of the EQ-5D-3L, and it seems that mobility, self-care and pain/discomfort were less important for non-specific LBP patients than for members of the general population. Both findings are in line with those of Mann et al. who attributed them to the possibility that non-specific LBP patients adapt to some extent to problems with mobility, self-care and pain/discomfort, but less to mental health problems [9].

### Strengths and limitations

The main strength of the present study is that it was the first to compare patient and population health state valuations among all subgroups of non-specific LBP patients. Herewith, the present study provides valuable input into the discussion of how to value health-related quality of life in cost-effectiveness analyses in general and in LBP research in particular.

The present study also has some limitations. First, the study population consisted of non-specific LBP patients who participated in a Dutch RCT. Consequently, it is unknown to what extent the present findings are generalizable to Dutch non-specific LBP patients in general, to those living outside the Netherlands, and to those suffering from LBP with a specific cause. Second, the present study relied heavily on VAS valuations, which are generally considered to be inferior to other health state valuation methods, such as the Time Tradeoff (TTO) and the Standard Gamble (SG) [6, 18]. Future research is, therefore, needed to explore whether the present findings would hold when using the TTO and the SG.

### Implications for research and practice

The present findings provide further evidence that it can make a difference whose health state valuations are used. That is, non-specific LBP patient valuations were statistically significantly and clinically meaningfully higher than population valuations. As a consequence, the incremental gain from reducing the participants’ complaints and restoring them to full health could have been 1.4 times larger when population instead of patient valuations were used [i.e., (1 − 0.731)/(1 − 0.632)]. This indicates that the choice of valuation method could have a substantial effect on the results of cost-effectiveness analyses [18]. Therefore, researchers are encouraged to explore the implications of the choice of valuation method on the outcome of their cost-effectiveness analysis using a sensitivity analysis. Second, the finding that only 22.2% of the variance in patient valuations was explained by the participants’ EQ-5D-3L health states indicates that the EQ-5D-3L might not be the optimal measure for estimating non-specific LBP patients’ self-perceived health-related quality of life. Therefore, further research is needed to explore which existing health state questionnaire (e.g., EQ-5D-3L, EQ-5D-5L, SF-6D, and HUI) is most suitable for estimating self-perceived health-related quality of life among non-specific LBP patients and/or whether an LBP-specific health state questionnaire ought to be developed.

## Conclusion

This study demonstrated that non-specific LBP patients value their health state better than members of the general population and that the choice of valuation method could have important implications for cost-effectiveness analyses and thus for healthcare decision making and clinical practice.

## Appendix 1: Frequencies and percentages with which the various EQ-5D-3L health states were scored

**Table:**

EQ-5D-3L Health state	Frequency	Percentage
11111	27	5.6
11112	2	0.4
11121	1	0.2
11211	12	2.5
11212	4	0.8
11221	2	0.4
11222	1	0.2
12111	50	10.4
12112	14	2.9
12121	2	0.4
12211	90	18.6
12212	73	15.1
12213	1	0.2
12221	16	3.3
12222	39	8.1
12311	3	0.6
12312	4	0.8
12321	4	0.8
12322	5	1.0
13111	1	0.2
13112	1	0.2
13212	9	1.9
13222	6	1.2
13312	2	0.4
13322	5	1.0
13333	1	0.2
21211	2	0.4
21212	1	0.2
21221	1	0.2
21222	2	0.4
22111	8	1.7
22112	1	0.2
22211	23	4.8
22212	23	4.8
22221	5	1.0
22222	12	2.5
22311	3	0.6
22312	3	0.6
22321	1	0.2
22322	2	0.4
23211	1	0.2
23212	5	1.0
23222	7	1.5
23322	3	0.6
32211	1	0.2
32322	1	0.2
33212	1	0.2
